# Development of chipless, wireless current sensor system based on giant magnetoimpedance magnetic sensor and surface acoustic wave transponder

**DOI:** 10.1038/s41598-018-20867-3

**Published:** 2018-02-05

**Authors:** Vijay V. Kondalkar, Xiang Li, Ikmo Park, Sang Sik Yang, Keekeun Lee

**Affiliations:** Department of Electrical and Computer Engineering, Ajou University, Woncheon-dong, Yeongtong-gu, Suwon 443–749 Republic of Korea

## Abstract

A chipless, wireless current sensor system was developed using a giant magnetoimpedance (GMI) magnetic sensor and one-port surface acoustic wave (SAW) reflective delay line for real-time power monitoring in a current-carrying conductor. The GMI sensor has a high-quality crystalline structure in each layer, which contributes to a high sensitivity and good linearity in a magnetic field of 3–16 Oe. A 400 MHz RF energy generated from the interdigital transducer (IDT)-type reflector on the one-port SAW delay line was used as an activation source for the GMI magnetic sensor. The one-port SAW delay line replaces the presently existing transceiver system, which is composed of thousands of transistors, thus enabling chipless and wireless operation. We confirmed a large variation in the amplitude of the SAW reflection peak with a change in the impedance of the GMI sensor caused by the current flow through the conductor. Good linearity and sensitivity of ~0.691 dB/A were observed for currents in the range 1–12 A. Coupling of Mode (COM) modeling and impedance matching analysis were also performed to predict the device performance in advance and these were compared with the experimental results.

## Introduction

In modern electrical technologies, there is a need for a system that monitors the real-time power usage at any location and then transmits the information wirelessly to a portable reader^1,2^. By monitoring the real-time power usage, unnecessary energy consumption can be prevented, and necessary actions can be taken to prevent any major accident in electric power distribution infrastructures^3–7^. When an electric car or a household appliance is being charged, there is a need for real-time monitoring of charging status; i.e., whether there is a proper flow of current or any contact failure or aging problem with the charger^8^. In order to realize such a system, current and voltage sensors with high sensitivity and reliability are necessary^9–12^. Variety of current sensors with different designs and operating mechanisms, which include resistive shunt current sensor, Hall sensor, Inductive sensor, Rogowski coil sensor, have been reported^13^. The resistive shunt sensor, which is based on Ohm’s law, measures the current through direct contact with the path of current. It is easy to measure DC and low frequency currents using this sensor; however, it requires complex signal-conditioning circuits and poses heat generation and safety issues^14,15^. Hall sensor is based on a non-contact method and is relatively safe. However, to measure external current, knowledge of both the magnetic field generated from current-carrying path and the current flowing in the sensor itself is required. In addition, it has problems with low breakdown voltage owing to the use of semiconductor and non-linearity in the measurement of high external currents. The Rogowski coil sensor has a copper wire wound around air or a non-magnetic core and measures the external current by using an integrator at the output terminal^16–18^. Large size, heaviness, high power consumption, and complex wireless communication technology are critical issues that need to be resolved^19^. Current sensors based on GMI and giant magnetoresistance (GMR) have been reported in literature, and are preferred because of their high sensitivity, wide frequency range, small size, low power consumption, and facile compatibility with CMOS technologies^20–25^. The development of this kind of current sensors has been facilitated by the progress in both theoretical and technological methods over several years. Compared to traditional sensors, the GMI and GMR sensors are compatible with many other state-of-the-art technologies^26,27^.

A wireless transceiver system is required to send the measured current to a remote reader. The currently existing wireless transceiver systems consist of a battery, an antenna, a voltage regulator, an amplifier, a de-modulator, a multiplexer, an A/D converter, and a digital processor^28–30^. These kinds of wireless systems have several issues including signal distortion and addition of noise when passing the wireless electronic circuits, and heavy re-chargeable battery installation to activate the circuits and current sensor. In addition, the currently existing wireless transceiver systems require signal processing to be performed twice. Signal processing is first fulfilled at the forefront of the wireless system and then again in the distant reader unit. Above all, these systems are expensive, heavy, high power consuming, and difficult to use when multiple sensors are integrated in a single system.

In the recent years, reflective surface acoustic wave devices have been implemented as passive transponders to overcome these drawbacks. Fu *et al*. reported wireless passive SAW sensor by using the single electrode-type IDT structure as reflector and is connected with the external capacitive and resistive sensors as load impedances^31^. Schimetta *et al*. developed a wireless pressure-measurement system with a combination of a SAW reflective delay line with a high-Q capacitive pressure sensor^32^. Li *et al*. integrated the magnetic fields, temperature, and humidity sensor with a SAW transducer to realize a passive and wireless multifunctional sensor^33^. Jung *et al*. developed a wireless neural probe in which the varicap diode interconnected with sharp metal shank was electrically linked to the SAW device^34^. Karilainen *et al*. developed the voltage sensor for biomedical application based on a SAW delay line with voltage-dependent impedance loading on a reflector IDT^35^.

**Figure 1:**
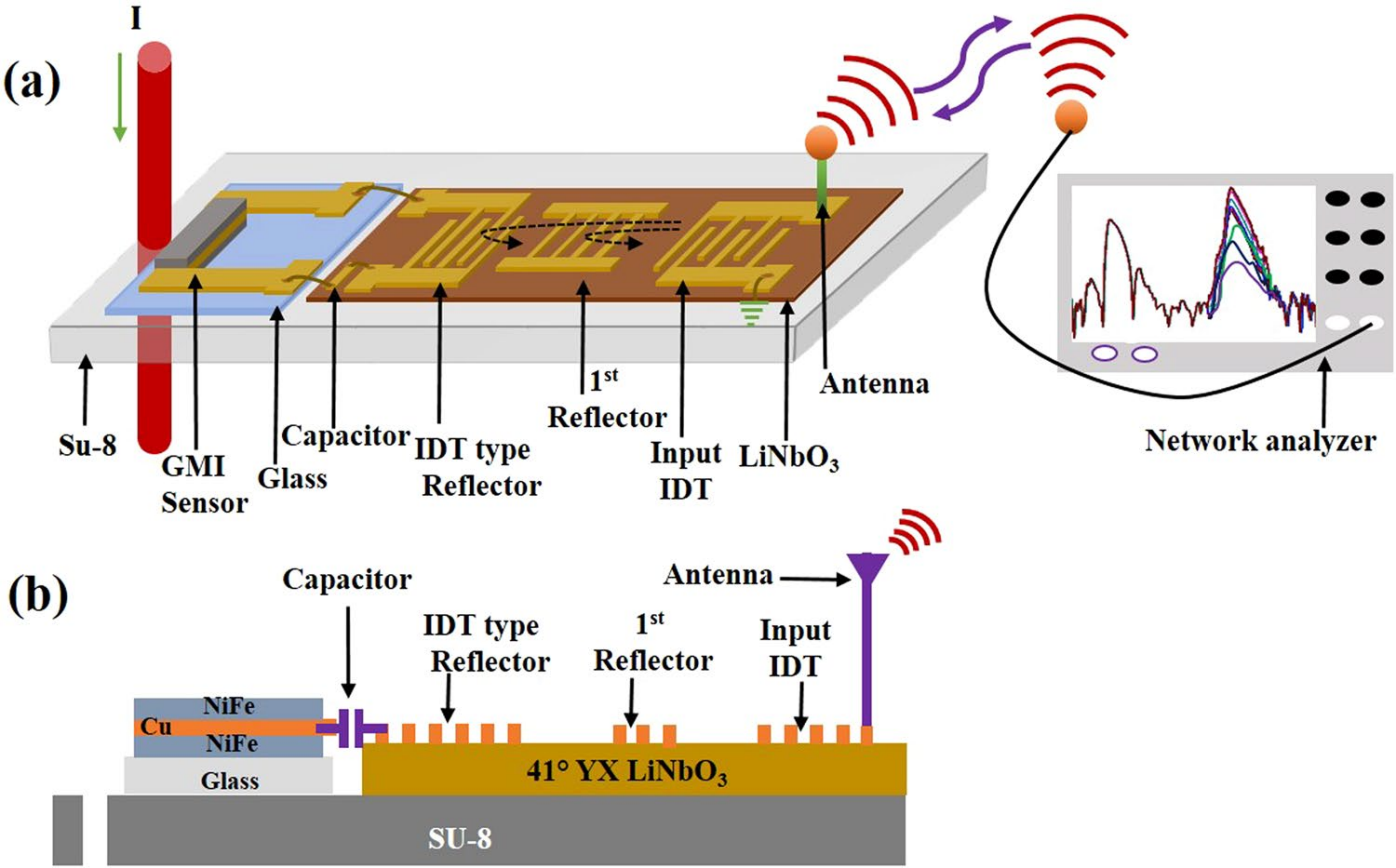
(**a**) Overall view of the developed chipless, wireless current sensor system. It consists of GMI sensor, one-port SAW delay line, impedance matching element, clip-on packaging platform, antenna, and reader system. (**b**) Cross-sectional view of sensor system on the clip-on packaging platform.

We present a chipless, wireless current sensing system that comprises of a GMI magnetic sensor and one-port surface acoustic wave (SAW) delay line. Figure 1 illustrates the overall view of the developed sensor and measurement system. The developed system consists of a GMI magnetic sensor for current sensing, one-port SAW delay line for the chipless and battery-free transceiver system, an impedance matching element for large sensitivity, two antennas, a clip-on packaging platform, and a network analyzer as a reader. When the IDT on the one-port SAW reflective delay line receives an RF energy through the antenna, a SAW generated on the piezoelectric substrate propagates towards the two reflectors. Some of the SAW energy is reflected by the reflectors, and returned to the input IDT and re-converted into EM wave by the transducer this EM wave is transmitted to the distant measurement system through the antenna. There are two reflection peaks at the reader system. The GMI magnetic sensor is connected to the second IDT-type reflector on the one-port SAW reflective delay line. The conductor line carrying the current produces a magnetic field that affects the impedance variation of the GMI; this gives rise to variations in the second reflection peak of the reader unit. By assessing the amplitude changes of the second reflection peak, the data related to the value of current in the conductor can be evaluated. This system possesses many merits over the currently existing wireless current sensor systems: it is chipless and battery-free so it does not require any complex transceiver systems and heavy re-chargeable battery to activate the circuits. The one-port SAW delay line supersedes the currently available transceiver system that consists of a few thousand transistors. A clip-on packaging platform was employed to control the distance, angle, and tilt between the current-carrying conductor and the GMI sensor. Coupling of mode (COM) modeling and impedance matching analysis were performed to predict the optimized structural parameters for the one-port SAW delay line and to make the change in amplitude of the second reflection peak large and linear in terms of the magnitude of the current flowing in the wire.

## Results and Discussion

### Design considerations and analytical modeling

#### Chipless, wireless SAW transponder

A one-port SAW reflective delay line was used to supersede the currently available wireless transceiver systems. The one-port SAW reflective delay line consists of input IDTs connected to an antenna, a shorted-grating reflector for a reference peak, and an IDT-type reflector linked to the GMI sensor. A change in impedance of the GMI sensor with respect to the external magnetic field gives rise to a variation in the reflection energy (${P}_{11}({Z}_{load})$) from the IDT-type reflector on the one-port SAW reflective delay line, according to the following equation^36,37^.

1 $${P}_{11}({Z}_{load})={P}_{11}^{sc}+\frac{2{P}_{13}^{2}}{{P}_{33}+\frac{1}{{Z}_{load}}}$$

Here, ${P}_{11}^{sc}$ is the acoustic reflection coefficient of the shorted-circuit IDT structure, ${P}_{13}$ is the electroacoustic coupling factor, ${P}_{33}$ is the electrical admittance of the IDT-type reflector, and ${Z}_{load}$ is the load impedance that includes the GMI and the impedance of the capacitor installed for impedance matching. The variations in ${P}_{11}({Z}_{load})$ caused by the external magnetic field result in a variation of the amplitude in the second reflection peak. By assessing the amplitude changes, the data about the current through the conductor line can be assessed.

COM modeling was performed to obtain the results for the one port SAW reflective delay line in advance. A 41° YX LiNbO_3_ piezoelectric substrate was chosen because it has a large electromechanical coefficient (*K*^2^ = 17%) and a high SAW velocity (~4000 m/s). The 400 MHz center frequency was selected, so that a reasonable antenna size could be used and also because the GMI sensor follows AC signals of ~400 MHz well. Thirty IDT finger pairs were selected and the aperture length was designed to be 40λ. Shorted grating bars were utilized for the reference reflector to induce higher reflectivity and lower insertion loss owing to the strong reflectivity and almost zero self-reflection, respectively. For the second reflector, an IDT-type reflector was chosen to convert the incoming SAW energy into AC signal for operating the GMI sensor.

**Figure 2:**
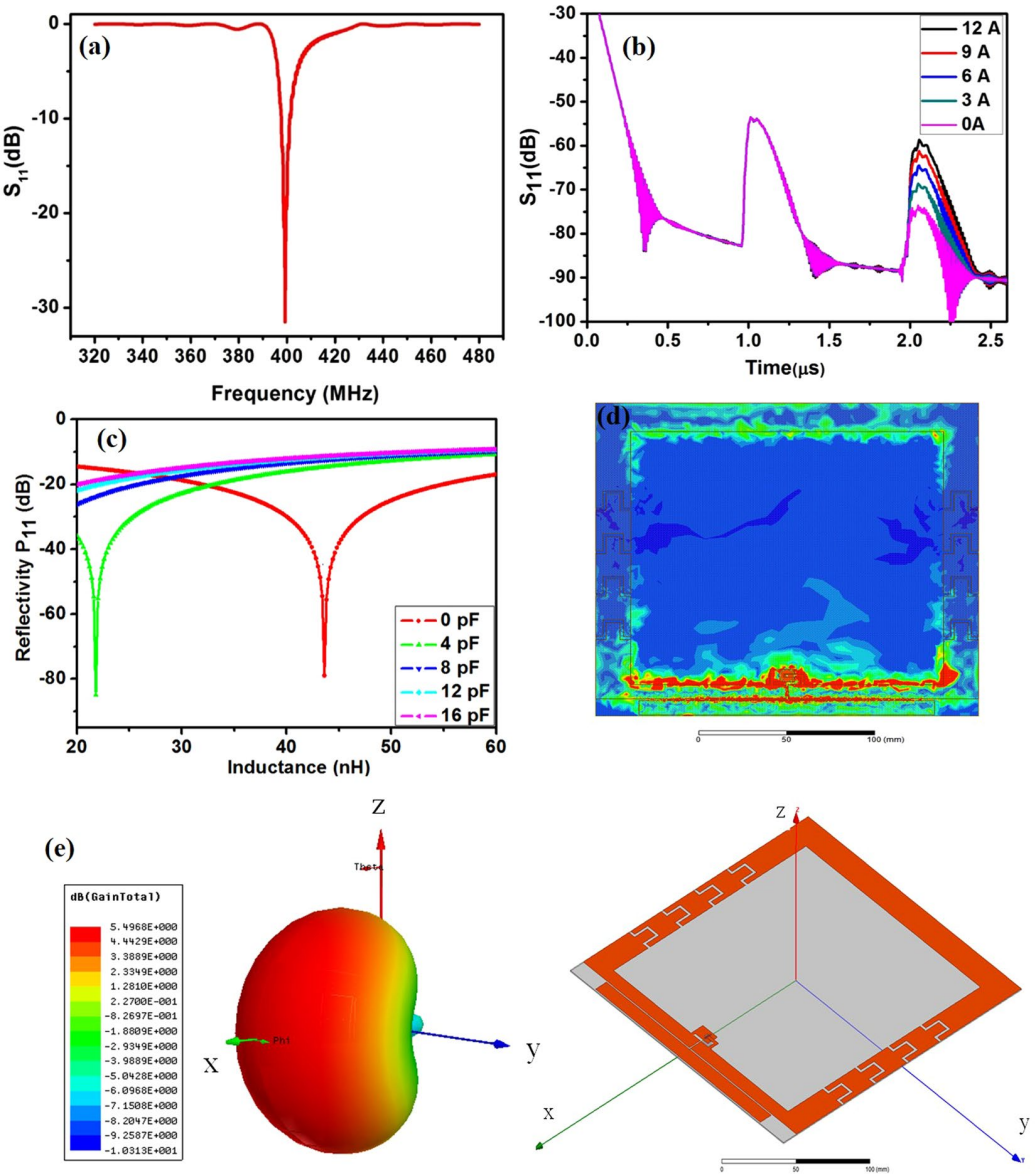
(**a**) Simulated S_11_ in terms of the freqeuncy for one-port SAW reflective delay line connected with GMI magnetic sensor, (**b**) Amplitude variations of the second reflection peak in terms of current flowing in conductor line, (**c**) Simulated results of the reflectivity of P_11_ as a function of the loads, (**d**) Surface current density on the patch array of the antenna at 400 MHz, (**e**) 3-D radiation pattern at 400 MHz.

Figure 2 shows the frequency and time response of the SAW delay line that were obtained by exploiting COM modeling. A sharp and narrow bandwidth peak was observed at 400 MHz center frequency region, as shown in Fig. 2(a), and two reflection peaks with high signal-to-noise ratio were observed in the time domain, as shown in Fig. 2(b). When the IDT-type reflector was simulated, the GMI sensor was treated as an inserted variable impedance source. Based on the disturbed impedance changes, the amplitude variations in the second reflection peak were extracted in terms of the external magnetic field generated by the current through the conductor, as shown in Fig. 2(b). COM simulations showed high sensitivity and good linearity in the amplitude variations.

To find the optimal capacitor in the system, simulation based on the following equation was carried out.

2 $${Z}_{load}=R+j\omega L(Hext)+1/j\omega {C}_{match}$$

Here, *R* is the average resistance of the sensor, and *L(H*_*ext*_) is the inductance of the GMI sensor in terms of the external magnetic field. Figure 2c shows the simulation results of the reflectivity of P_11_ as a function of the Z_load_. The GMI sensor has an inductance range of 20–44 nH. From among the various capacitors, an 8 pF capacitor was chosen as the matching element and was connected in series with the GMI sensor owing to its highest reflectivity and good linearity in terms of the inductance. The ~8 pF capacitor was selected as the best matching element for different magnetic fields. The slope of the plot indicates the magnetic field sensitivity. With the optimal matching capacitance, a maximum reflectivity change rate of 0.67 dB/nH was observed.

#### Antenna

The surface current density in the patches was calculated to explain the radiation mechanism (Fig. 2d). Only the patches along the slit line were activated and they had a strong current across the direction of the slit line (y-direction) that produced a strong field in the perpendicular direction. This outcome resulted in a wide beam width in the xz-plane and the antenna showed low back radiation. The antenna acts as a Yagi antenna with a director. Thus, the antenna radiates the majority of power in the direction of the strip. Figure 2e shows the 3-D radiation pattern of the antenna with the reference coordinate.

### Fabricated devices

**Figure 3:**
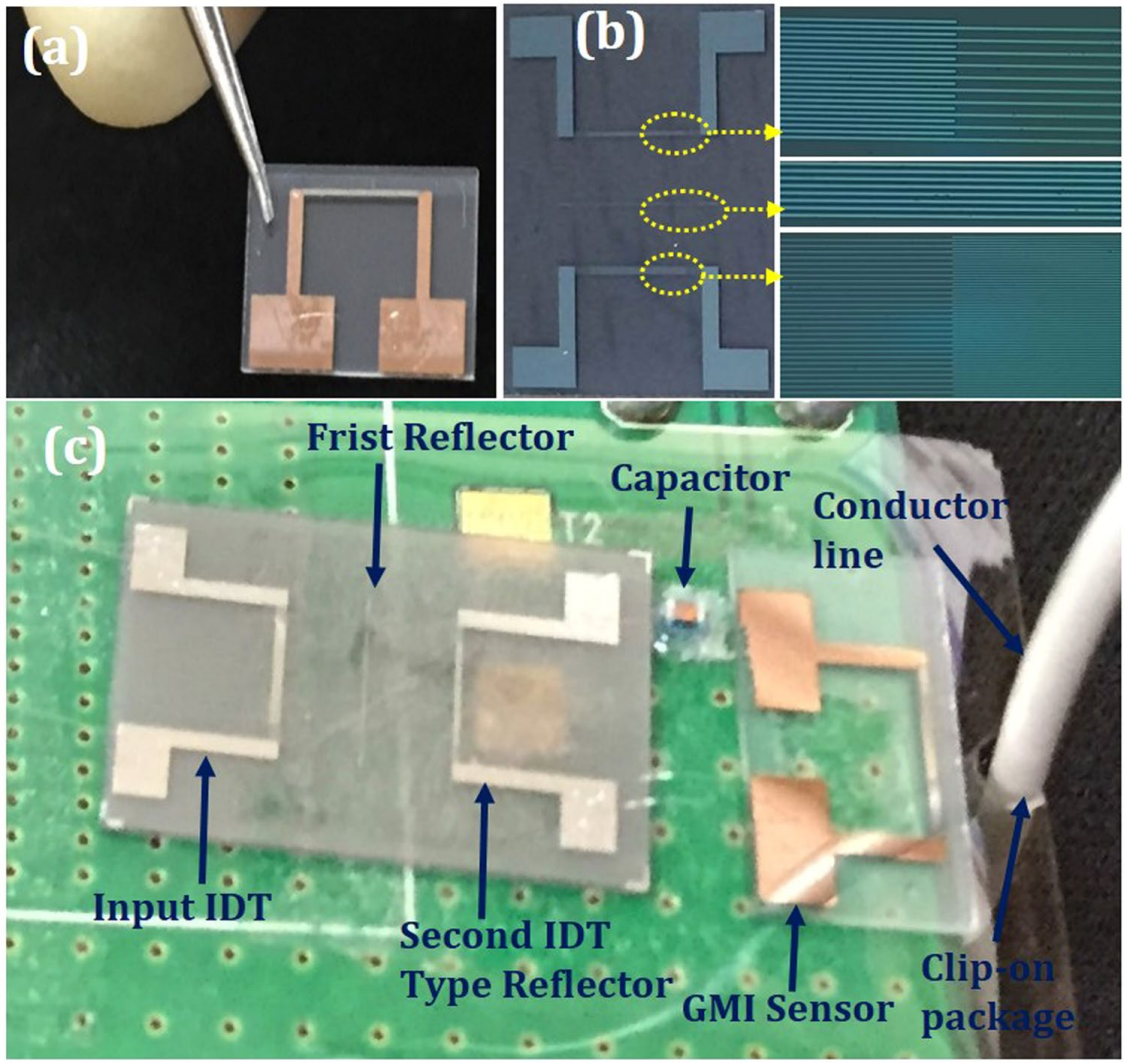
(**a**) View of the fabricated GMI sensor, (**b**) Overall and magnified views of the one port SAW reflective delay line, (**c**) Assembled units of all the devcies on clip-on packaging platform for wireless current sensing.

Figure 3 shows an overall view of the fabricated devices. For the GMI sensor, Cu was sandwiched between two soft magnetic material (NiFe) layers. The thickness of NiFe and Cu were 200 nm and 100 nm, respectively. The fabricated GMI sensor is shown in Fig. 3(a). The length and width of the GMI sensor along the stripe line were 2 mm and ~150 μm, respectively. Figure 3(b) shows the one-port SAW reflective delay line composed of the input IDT, shorted grating reflector, and IDT-type second reflector. For the input IDT, the number of finger pairs was 30, the finger width was 2.5 μm, and the aperture length was 40λ. The first shorted-grating reflector was positioned 2.2 mm away from the input IDTs. The distance between the second reflector and the input IDT was ~4.6 mm. Figure 3(c) shows an overall view of the assembled units. The whole system is fixed to a conductor line with clip-on packaging platform and is secured to maintain the distance, angle, and tilt between the current-carrying conductor line and the GMI sensor.

### GMI sensor

**Figure 4:**
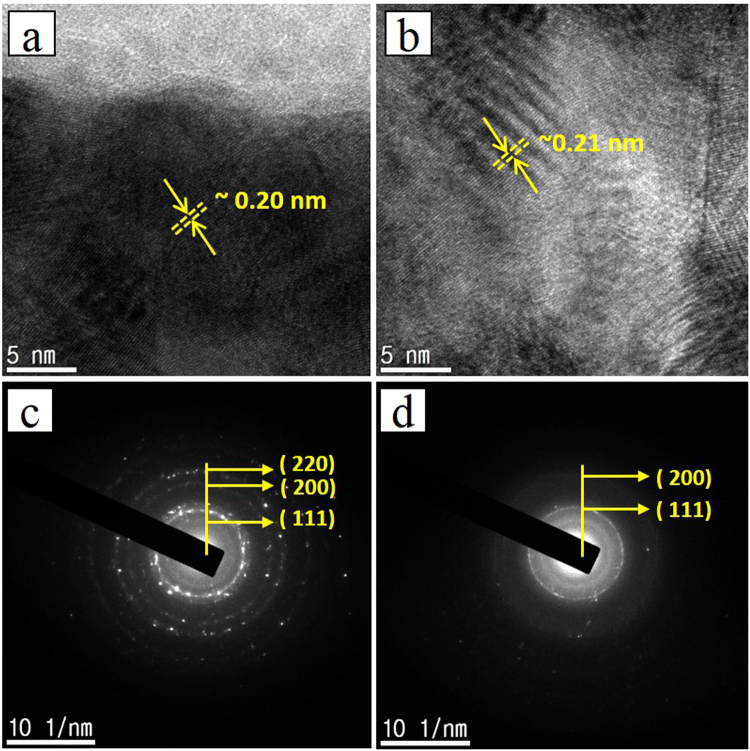
(**a**) HRTEM image of NiFe, (**b**) HRTEM image of Cu, (**c**) SAED pattern of NiFe, (**d**) SAED pattern of Cu.

**Figure 5:**
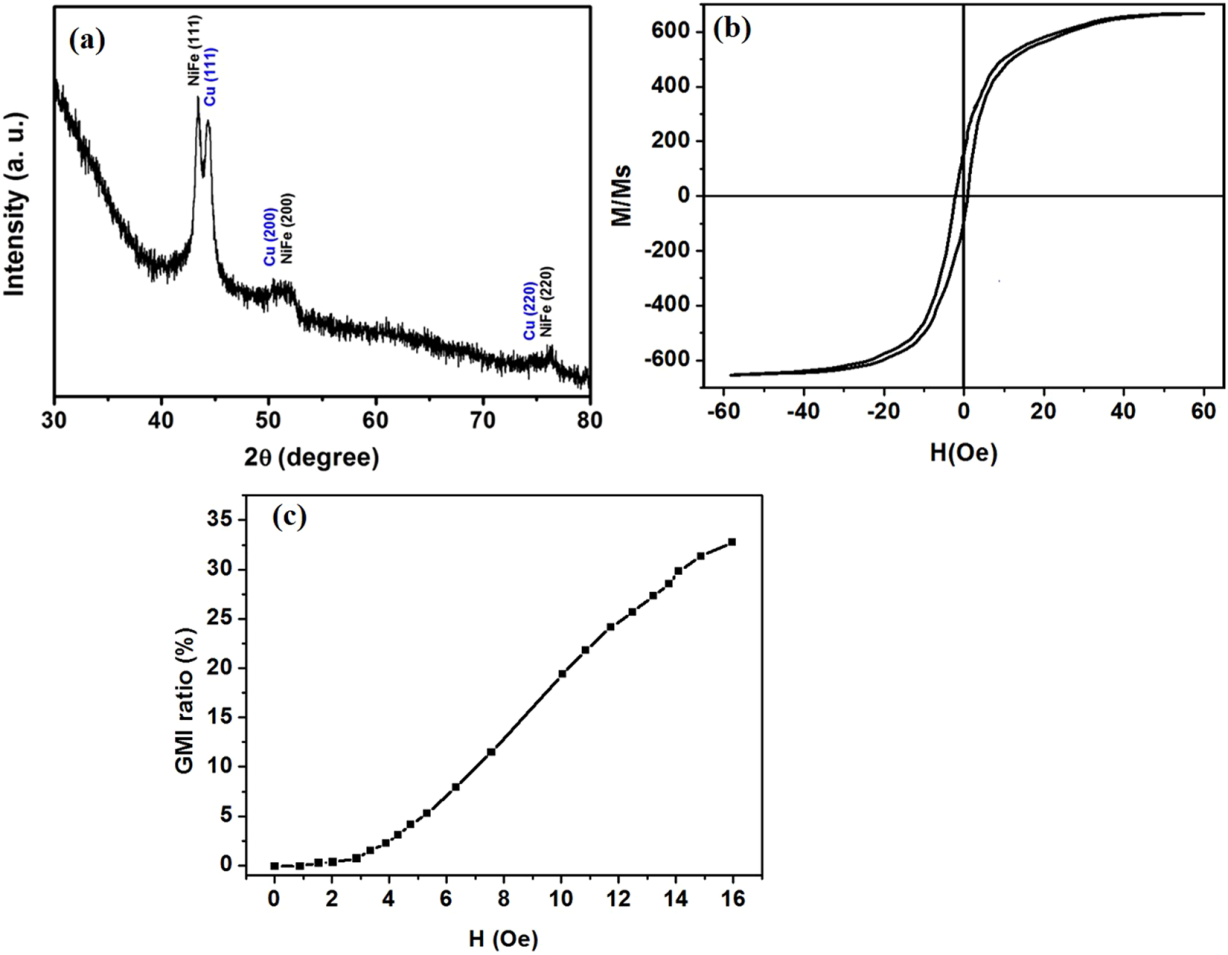
(**a**) X-ray diffraction pattern of the fabricated GMI magnetic sensor, (**b**) B-H curve of the GMI sensor, (**c**) Variation of GMI as a function of magnetic field.

The high resolution transmission electron microscopy (HRTEM) analysis of the fabricated GMI sensor was performed to analyze the structural features as shown in Fig. 4(a,b). The HRTEM image reveals that the nanoparticles are densely distributed over the entire region in the NiFe and Cu layer. Figure 4a demonstrates that the lattice fringe spacing is about 0.20 nm in the NiFe layer, which is well indexed to the (111) plane of the cubic crystal structure of NiFe. The fringe spacing in the Cu layer was ~0.21 nm (Fig. 4b) which also well matched with the (111) plane of the cubic crystalline structure of Cu. The selected area electron diffraction (SAED) pattern displayed in Fig. 4(c,d) suggested a high crystal quality with a nanocrystalline nature of sputtered NiFe and Cu. Furthermore, the phase purity and crystal structure of the NiFe and Cu in the GMI device were investigated using the X-ray diffraction pattern. As shown in Fig. 5(a), the diffraction peaks of NiFe well agreed with the indices of the (111), (200), and (220) planes corresponding to the cubic crystal structure of NiFe, and the Cu diffraction peaks well agreed with the indices of the (111), (200), and (220) planes corresponding to the cubic crystal structure. The coercive forces and anisotropy field along the longitudinal magnetization direction were obtained by measuring the B-H curve. Figure 5(b) reveals a low coercive force, high anisotropy field, and low saturation induction indicating that the grown NiFe has a desirable soft ferromagnetic behavior. The coercive field strength (Hc) was ~1.28 Oe. The fabricated GMI sensor has a well-defined transverse magnetic anisotropy. In order to analyze the performance of the GMI sensor, a magnetic field H_ex_ was applied along its longitudinal direction. The magnetic field response of the GMI sensor was measured by an impedance analyzer shown in Fig. 5c. The GMI ratio was calculated from Z(H) curves defined in the equation (3) below:

3 $$GMI\,ratio=[Z({H}_{ex})-Z({H}_{0})]/Z({H}_{0})\times 100 \% $$

where Z(H_ex_) and Z(H_0_) are the magnetoimpedances in the presence and absence of an external magnetic field, respectively. The magnitude of the GMI increases up to 32.94% at 16 Oe. Further, sensitivity was calculated using the equation (4),

4 $$Sensitivity=[Z({H}_{2})-Z({H}_{1})/Z({H}_{1})]/({H}_{2})-({H}_{1})\times 100 \% $$

The sensitivity was found to be 3.23%/Oe in magnetic fields of strength 3–16 Oe. The high sensitivity observed in the lower magnetic field region in the thin film GMI sensor makes it a better candidate for low-field magnetic sensing applications.

### Chipless SAW transponder

**Figure 6:**
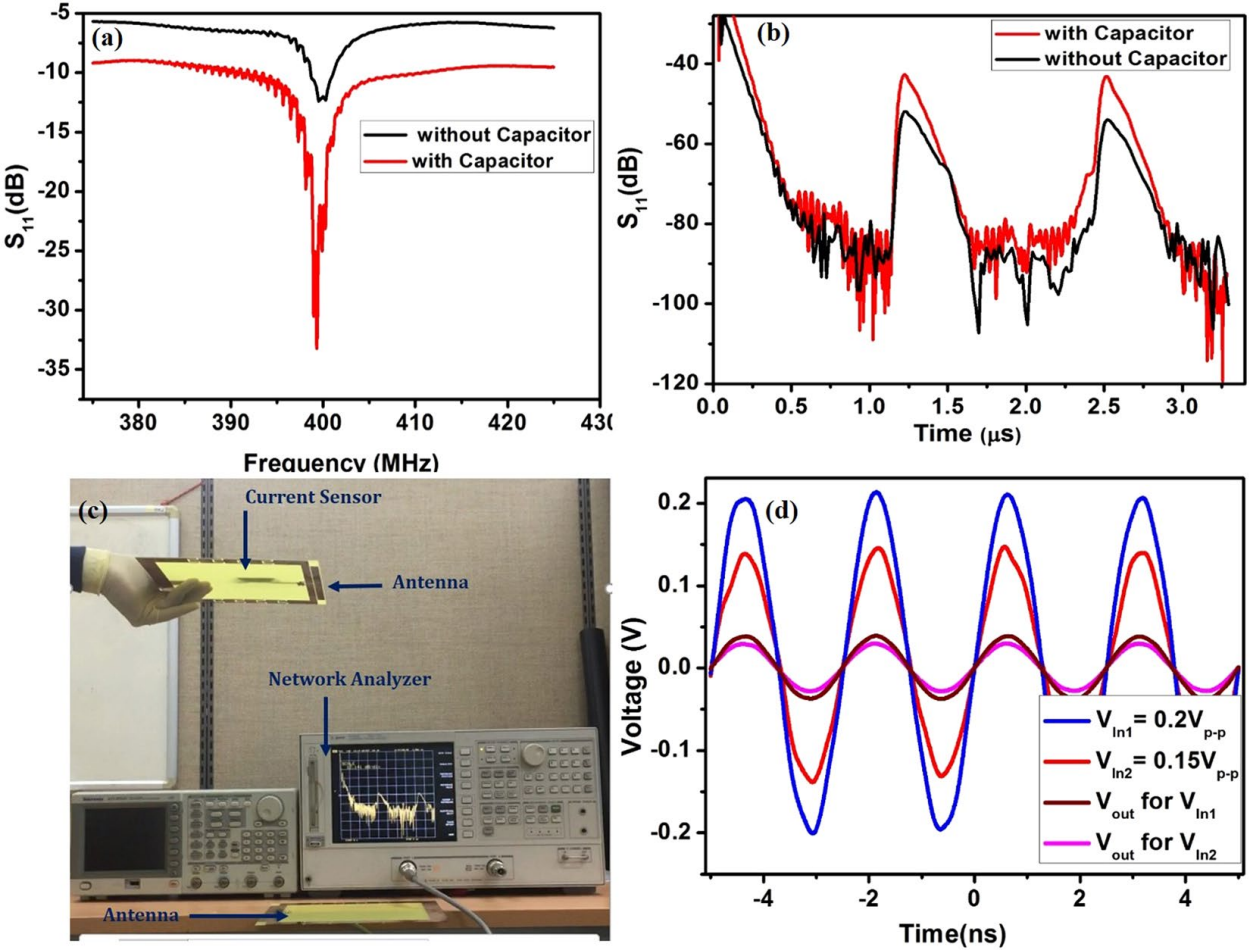
S_11_ response for one-port SAW reflective delay line with and without capacitor for impedance matching in (**a**) the frequency domain, (**b**) the time domain, (**c**) Wireless readout distance testing in terms of RF powers from the network anlayazer, (**d**) AC voltages obtained from the second IDT-type reflector in terms of different input peak-to-peak voltages.

Two antennas with center frequency of ~400 MHz were developed. One antenna was connected to the network analyzer while the other was connected to the input IDT of the SAW reflective delay line. The input RF power of ~1 mW was swept from 300 to 500 MHz, and the reflective scattering parameter S_11_ was measured in the frequency and time domains. A center frequency of 400 MHz was observed in the frequency domain (Fig. 6(a)), and S_11_ was sharp and had a narrow bandwidth. The experimental results were in good agreement with the COM modeling predictions with respect to the 400 MHz center frequency and the response shape. The reflective scattering parameter S_11_ obtained in the frequency domain was converted into the time domain as shown in Fig. 6(b). Two reflection peaks were observed with a large signal-to-noise ratio. The first reflection peak was observed at ~1.2 μs, which was used as the reference peak. The second reflection peak appeared at 2.3 μs and was connected to the GMI sensor via the impedance matching element. All the reflected peaks agreed well with the predicted values from the COM modeling in the frequency and time domain. The SAW delay line with and without the impedance matching element (8 pF capacitor) was compared to verify whether the impedance matching element lowers the insertion loss and increases the amplitude of the reflection peak. The second IDT-type reflector on the SAW delay line was linked to the GMI sensor by the 8 pF capacitor in the middle. As shown in Fig. 6(a,b), the SAW delay line with the impedance matching element showed a lower insertion loss and higher signal to noise ratio when compared to the one without the impedance matching element. The center frequency and time positions of the reflectors were not changed, which is desirable for implementing the wireless, chipless current sensor system. The RF power from the network analyzer was changed to test the maximum reading distance (Fig. 6c). An increase in the applied RF power introduced a corresponding increase in the reading distance. With an RF power of 10 mW from the network analyzer, a readout distance of 5 m was observed. This reading distance can be further increased up to ~10 m by measuring the device under an EM shielded environment as given in ESI.

The GMI sensor was driven by AC signals generated from the second IDT-type reflector on the SAW reflective delay line; the IDT-type reflector converts the incoming SAW energy into AC signals. The AC voltages generated by the second IDT-type reflector were monitored using a high-frequency oscilloscope (Fig. 6d). A constant RF energy of 400 MHz was applied to the input IDT on the SAW delay line from a signal generator and the resultant output voltages at the IDT-type reflector were measured. AC signals with a range of ~0.05 V_peak-to-peak_ were observed at the IDT-type reflector, which was used as the activation source for the GMI sensor. With an increase in the RF input power, there was a corresponding increase in the output peak-to-peak voltage, without any change in the frequency (Fig. 6d).

### Wireless current sensing

**Figure 7:**
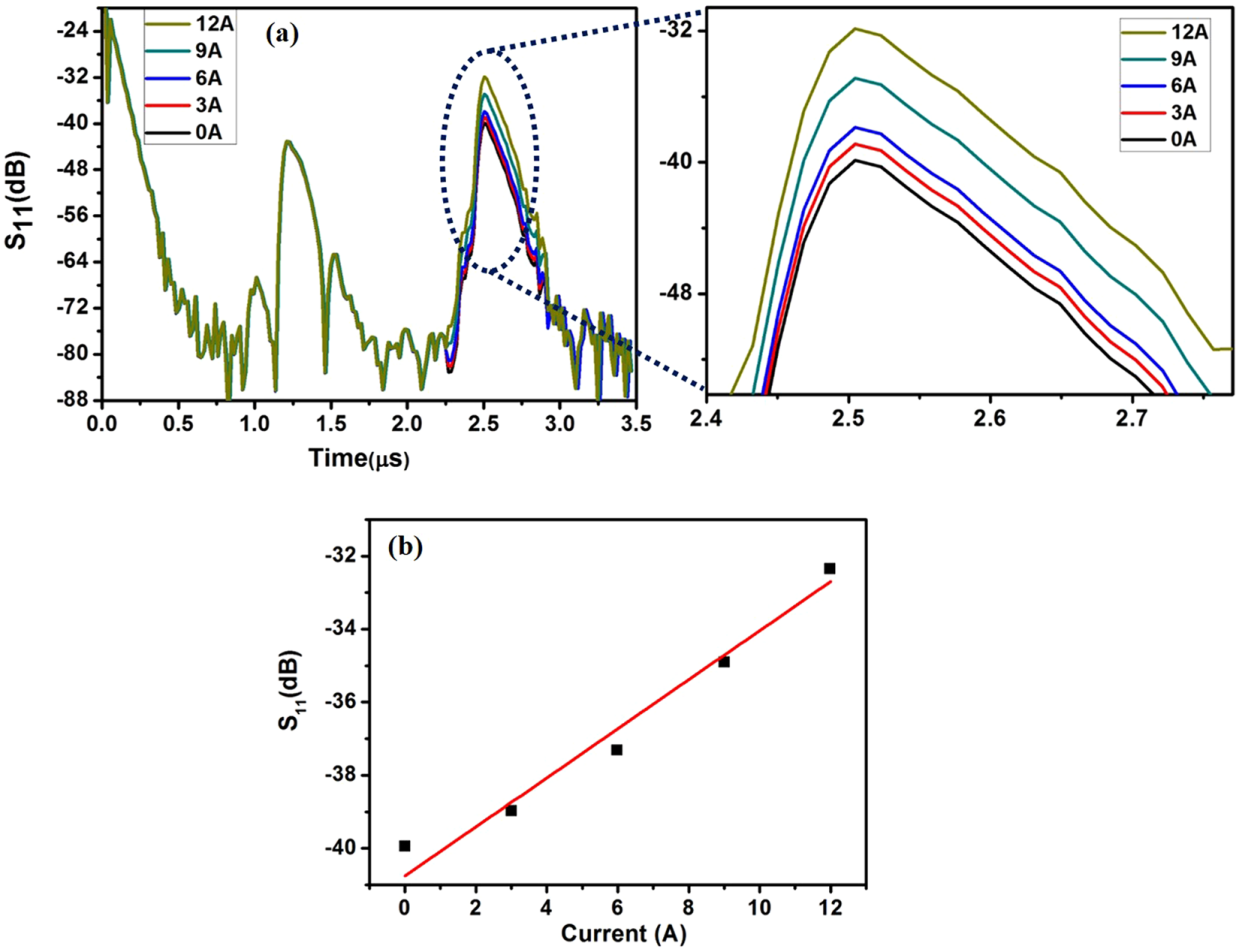
(**a**) Amplitude variations of the second reflection peak with respect to current flowing through conductor, (**b**) Calibrated amplitude variation of the sensor in terms of current flow in conductor line.

A conducting line and the GMI sensor were fixed with the thick SU-8 polymer based clip-on packaging platform. The distance between the GMI sensor and conductor line was maintained at 2 mm. A constant DC of 1 A was passed through the conductor. The amplitude variation at the second reflection peak was observed due to the magnetic field generated by the flow of current that creates a magnetic spin orientation within the GMI device, leading to a change in the load impedance Z_load_. Figure 7a shows a variation in the amplitude of the second reflection peak with an increase in the current through the conductor. The amplitude of S_11_ varied from −40.03 dB to −32.34 dB when the current through the conductor was increased from 1 A to 12 A. Here, the reading distance between the antennas and the input RF power were maintained constant as these parameters also affect the amplitude of the second reflection peak. The setting of a reference point at the amplitude of the second peak depends on the amplitude of the first reflection peak. The amplitude of the first peak was predicted based on the reading distance and the input RF power and the reference point at the amplitude of the second peak was determined based on the amplitude of the first peak. The variations in amplitude of the second reflection peak were re-plotted to obtain the sensitivity in terms of the current through the conductor, as shown in Fig. 7b. Calibration was conducted to find the linearity and sensitivity. The sensitivity (*S*) of a sensor can be obtained using the equation (5)

5 $$S={\rm{\Delta }}{S}_{11}(dB)/({\rm{\Delta }}A).$$

The highest sensitivity 0.691 dB/A was obtained and the linearity was found to be 0.962. The amplitude variations in the time domain, depending on the external current flow, were clearly observed; this is a promising result for the development of the battery-free, wireless sensor system.

## Conclusions

This work creates a solid foundation for the development of a new configuration of current sensor system. For the first time, wireless current sensor system without transceiver system and interface circuitries were successfully designed, fabricated, and tested. This senor system can eliminate currently complicated transceiver systems composed of thousands of electronic components and rechargeable heavy battery and can make battery-free, wireless measurements possible. A theoretical model for the prediction of a SAW amplitude variation with respect to the current flowing in the conductor was validated experimentally. A large variation in the amplitude of the reflection coefficient S_11_ was observed when the conductor carried a current. A sensitivity of 0.691 dB/A and a good linearity were observed for currents in the range 1–12 A. Our future work will be focused on integrating a voltage sensor with the current sensing system for monitoring the real-time power in a conductor.

## Methods

### GMI Sensor

**Figure 8:**
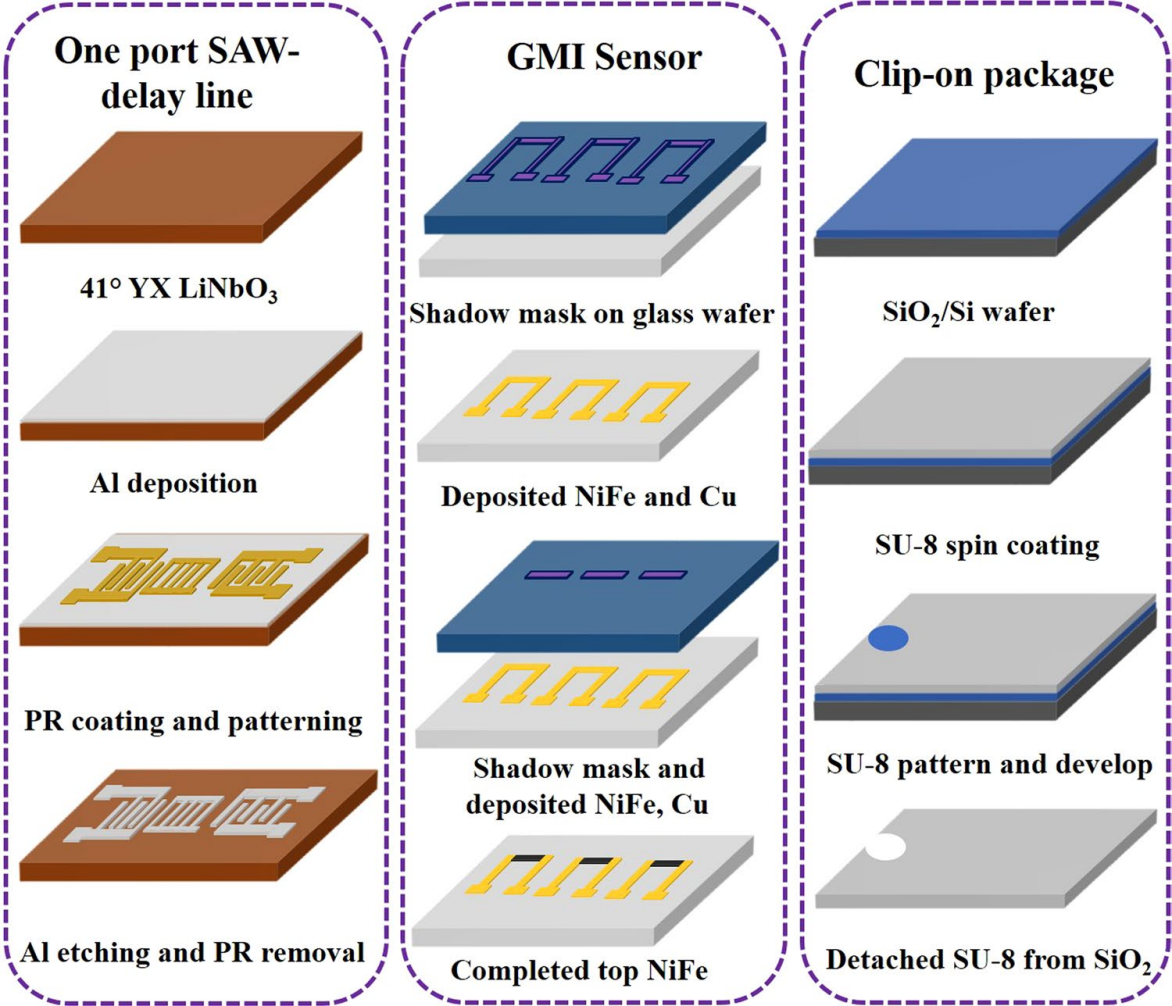
Schematic views of the fabrication procedures for one-port SAW reflective delay line, GMI sensor, and clip-on packaging platform.

A GMI sensor was developed on glass substrate using a DC sputter system. The magnetic alloy layer (NiFe) and the conducting layer (Cu) were deposited from elemental Ni_80_Fe_20_ and Cu targets respectively. The base pressure before sputtering was dropped to ~1.5 × 10^−6^ torr and the Ar gas pressure during the sputtering process was maintained at 3.5 × 10^−3^ torr. Each of the NiFe and Cu layers were sputtered successively from the respective targets under the same Ar gas pressure, and the deposition rates for NiFe and Cu were 2.0 Å/s and 5.0 Å/s, respectively. A magnetic field of 150 Oe was applied perpendicular to the stripe length of the substrate holder during deposition to obtain a desirable magnetic anisotropy. For the desired width (150 μm) and length (2 mm) of the GMI sensor, a shadow mask was used during the sputtering process. The thicknesses of the NiFe and Cu layer were 200 nm and 100 nm, respectively. Schematic view of the fabrication of the GMI sensor via sputtering is shown in Fig. 8.

### SAW transponder

A 400 MHz one-port SAW reflective delay line was developed using the CMOS process. A 250 nm thick Aluminum (Al) layer was deposited on a 41° YX LiNbO_3_ piezoelectric substrate using an e-beam evaporator and a 1 µm thick photoresist was then spin-coated on the wafer which was subsequently exposed and developed for the SAW transponder. The aluminum was wet-etched in a 4H_3_PO_4_:1HNO_3_:4CH_3_COOH:1H_2_O solution and then the photoresist was removed in microstriper. It was rinsed several times with deionized water to eliminate the unwanted particles. The final devices were dicing-sawed to obtain the individual device, which were then wire-bonded with antenna pads. The detailed process for the fabrication of SAW devices is shown in Fig. 8.

### Clip-on package platform

A clip-on package platform was fabricated using a thick SU-8 polymer to control the distance, angle, and tilt between the current-carrying conductor and the GMI sensor, as shown in Fig. 1. SU-8 polymer was spin-coated on a clean SiO_2_/Si wafer to obtain a thickness of ~100 μm. The coated SU-8 polymer was soft-baked at 65 °C for 30 min and then at 95 °C for 10 min. Next, the SU-8 polymer layer was UV-exposed, post-baked at 65 °C for 15 min and then at 95 °C for 5 min. It was later developed to get a desired hole on the SU-8 platform. The ~100 μm thick flexible SU-8 polymer was detached from the SiO_2_ wafer using buffered oxide etch (BOE) solution. The diameters of the holes were varied depending on the diameter of the conductor wire to be used.

### Antenna and Unit assembly

A two-dimensional planar loop antenna with 400 MHz central frequency was fabricated using an 8-mm-thick FR4 substrate (dielectric constant k = 3.38). More details of antenna design are given in ESI. All the separately fabricated devices were put together to construct the chipless, wireless current sensor system. First, on the clip-on package platform, the GMI device, one-port-SAW reflective delay line, and the capacitor were assembled. The spacing between the center of the hole and the GMI sensor was maintained at 2 mm. The GMI was electrically connected to the corresponding IDT-type reflectors on the one-port SAW reflective delay line via an 8 pF capacitor in the middle. For wireless measurements, the two antennas were connected to the S_11_ port of the network analyzer and the pads of the one-port SAW delay line. A conductor wire was clamped to the hole on the SU-8 package platform. The reflection coefficient S_11_ was wirelessly measured in the time domain.

## Electronic supplementary material

Supplementary Information
