# Supplementary material for: Development of chipless, wireless current sensor system based on giant magnetoimpedance magnetic sensor and surface acoustic wave transponder
Source: Sci Rep. 2018 Feb 5;8:2401. doi: 10.1038/s41598-018-20867-3 (PMC5799300; doi:10.1038/s41598-018-20867-3)
Supplement: Supplementary file 1 — Supplementary Information [file 41598_2018_20867_MOESM1_ESM.docx]

**Electronic Supplementary Information(ESI)**

**Development of chipless, wireless current sensor system based on giant magnetoresistance magnetic sensor and surface acoustic wave transponder**

Vijay V. Kondalkar^1^, Xiang Li^1^, Ikmo Park^1^, Sang Sik Yang^1^, and Keekeun Lee^1^*

^1^Department of Electrical and Computer Engineering, Ajou University, Woncheon-dong, Yeongtong-gu, Suwon 443-749, Republic of Korea,

E-mail: [keekeun@ajou.ac.kr](mailto:keekeun@ajou.ac.kr)

The antenna design is based on the rectangular loop partially loaded with mu-negative metamaterial unit cells [1]. The original antenna has its first resonance at around 670 MHz. We have rescaled the antenna to have its first resonance at around 400 MHz. The geometry of the antenna is shown in Fig. S1 and its fabricated antenna in Fig. S2. The antenna is printed on a single layer of a square FR4 substrate with side length of *L*=220 mm, thickness of 0.8 mm, dielectric constant of 4.4, and loss tangent of 0.02.


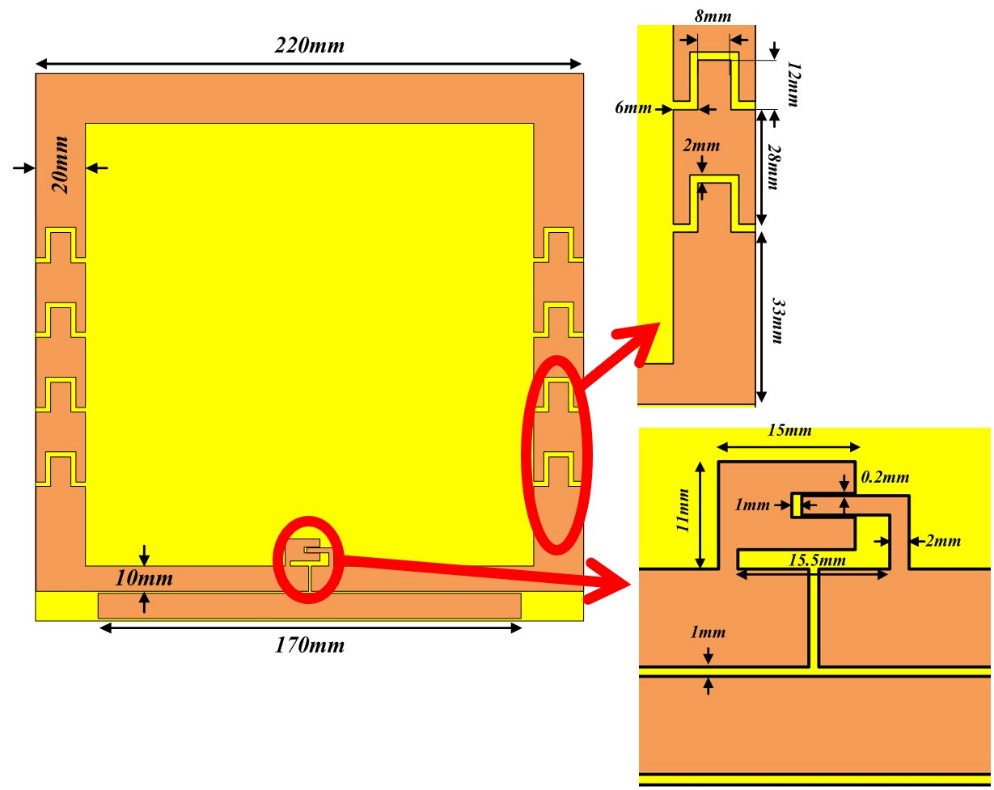


Figure. S1. Geometry of the antenna


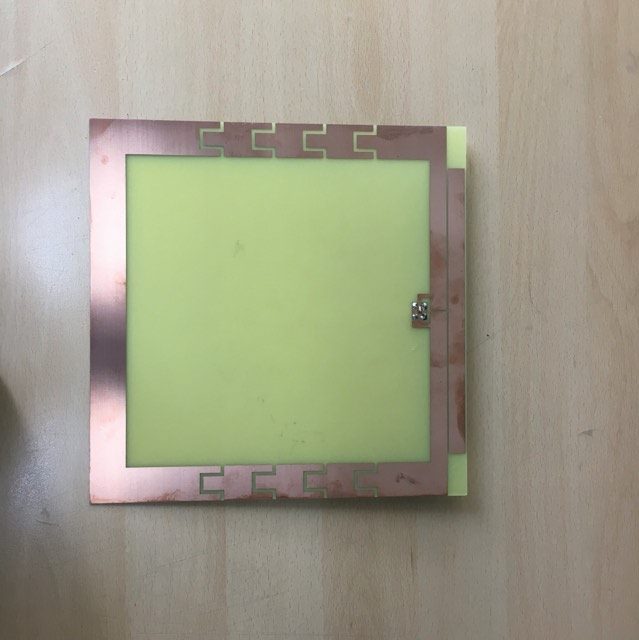


Figure. S2. Fabricated antenna

1. (b)

Figure. S3. Radiation patterns of the antenna at 400 MHz: (a) x-z plane and (b) y-z plane.

Fig. S3 presents the radiation patterns of the antennas at 400 MHz frequency. The antenna showed clean profile radiation patterns with low side lobe levels and back radiation in both the *xz*-plane and *yz*-plane at the investigated frequency. The antenna produced directive radiation patterns in the *yz*-plane, while the antenna showed radiation patterns with a wider beamwidth in the *xz*-plane. The half power beam width (HPBW) was approximately 90º for antenna. The HPBW in the xz-plane increased while having a narrow HPBW in the yz-plane. Notably, the strong current flows across the slit line (y-direction) produced a strong field in the perpendicular direction that resulted in a wider beamwidth in the xz-plane. The side lobe levels in both principal planes were very small. The 5.3dBi gain in broadside direction, 0.1dBi gain in backside direction.


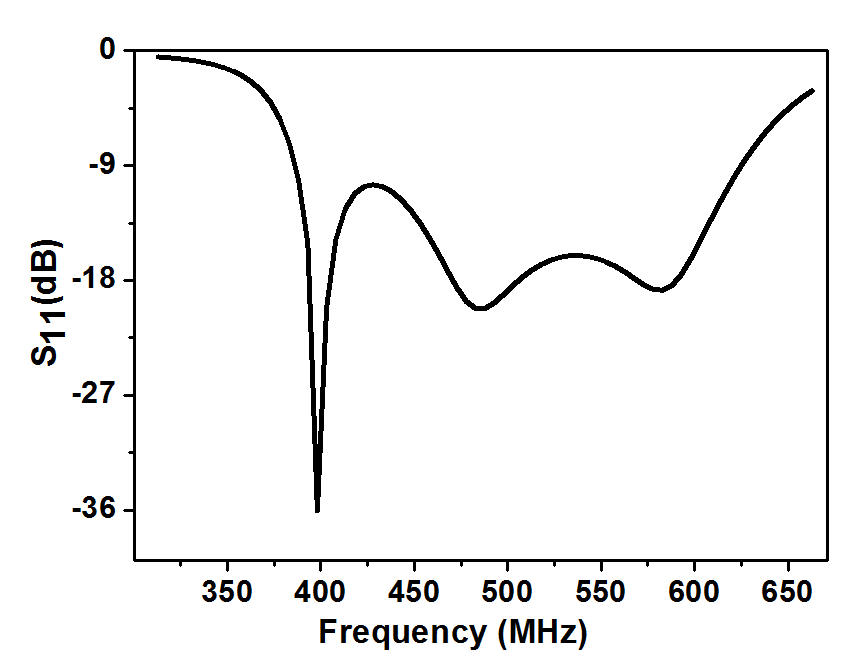


Figure. S4. Reflection coefficient of the antenna.

To reduce the electrical size of the antenna, its first resonance is formed by capacitively loading a conventional one wavelength loop antenna to excite the mu-zero resonance that is independent of the resonator’s size. To enhance the bandwidth of the antenna at lower frequencies and excite an additional resonance, a strip patch is added in the vicinity of the loop. The antenna shows wideband characteristics as shown in Fig. S4. The impedance bandwidth for |S11| < -10 dB => 373MHz~604MHz is 231MHz (57.7%). With its advantages of simple configuration and wideband operation, the proposed design is a good candidate for use in wireless communication systems. Further reading distance can be increased up to ~10 m by measuring the sensor device under an EM shielded environment shown in Fig. S5. The S/N ratio at different distance are given in table 1.


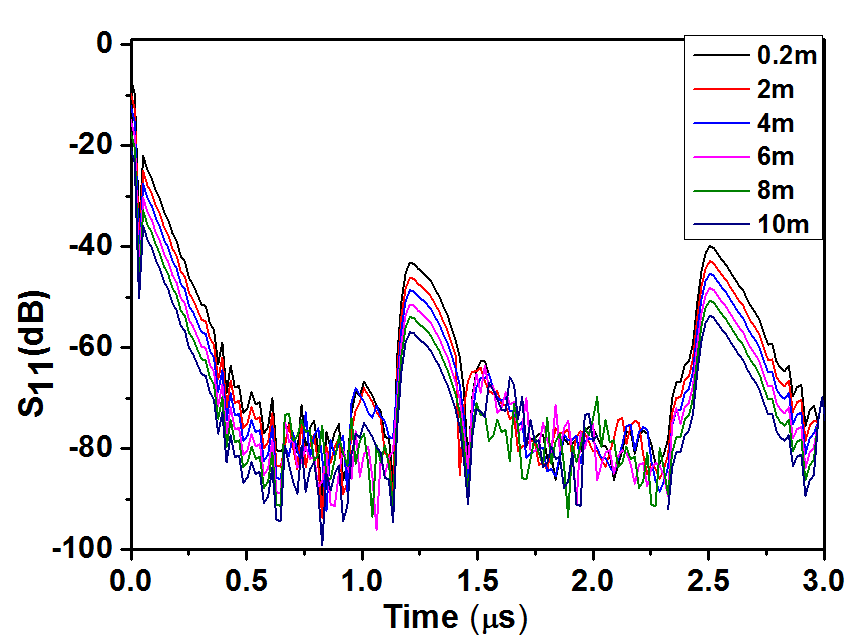


Figure. S5. S parameter performance of sensor at different distance

Table.1 S/N ratio at different distance

| Distance (m) | S/N (dB) |
| --- | --- |
| 0.2 | 10.71 |
| 2 | 10.36 |
| 4 | 8.01 |
| 6 | 6.69 |
| 8 | 5.21 |
| 10 | 3.23 |

**Reference**

1. Rezaeieh, S. A., Antoniades, M. A. and Abbosh, A. M. “Compact wideband loop antenna partially loaded with mu-negative metamaterial unit cells for directivity enhancement,” *IEEE Antennas Wireless Propag. Lett*., **15**, 1893, doi: 10.1109/LAWP.2016.2542799 (2016).
